# Structural basis for haem piracy from host haemopexin by *Haemophilus influenzae*

**DOI:** 10.1038/ncomms11590

**Published:** 2016-05-18

**Authors:** Silvia Zambolin, Bernard Clantin, Mohamed Chami, Sylviane Hoos, Ahmed Haouz, Vincent Villeret, Philippe Delepelaire

**Affiliations:** 1 CNRS, Université Paris 7 UMR 7099, Institut de Biologie Physico-Chimique, 13 rue Pierre et Marie Curie, 75005 Paris, France; 2 Université de Lille, CNRS, UMR 8576 - UGSF, Unité de Glycobiologie Structurale et Fonctionnelle, Laboratoire de Biologie Structurale Intégrative, F 59000 Lille, France; 3 Center for Cellular Imaging and Nano Analytics (C-CINA), Biozentrum, University of Basel, Mattenstrasse 26, CH-4058 Basel, Switzerland; 4 Institut Pasteur, Plate-forme de Biophysique, CNRS-UMR 3528, 25, rue du Dr Roux, 75724 Paris Cedex 15, France; 5 Institut Pasteur, Plate-forme de Cristallographie, CNRS-UMR 3528, 25, rue du Dr Roux, 75724 Paris Cedex 15, France

## Abstract

*Haemophilus influenzae* is an obligate human commensal/pathogen that requires haem for survival and can acquire it from several host haemoproteins, including haemopexin. The haem transport system from haem-haemopexin consists of HxuC, a haem receptor, and the two-partner-secretion system HxuB/HxuA. HxuA, which is exposed at the cell surface, is strictly required for haem acquisition from haemopexin. HxuA forms complexes with haem-haemopexin, leading to haem release and its capture by HxuC. The key question is how HxuA liberates haem from haemopexin. Here, we solve crystal structures of HxuA alone, and HxuA in complex with the N-terminal domain of haemopexin. A rational basis for the release of haem from haem-haemopexin is derived from both *in vivo* and *in vitro* studies. HxuA acts as a wedge that destabilizes the two-domains structure of haemopexin with a mobile loop on HxuA that favours haem ejection by redirecting key residues in the haem-binding pocket of haemopexin.

## Abstract (web summary)

Haemophilus influenzae requires haem, and acquires it from host haemoproteins including haemopexin. Here, the authors examine the haem transport system consisting of HxuA, HxuB and HxuC via the structures of HxuA in complex with haemopexin.

## Graphical abstract

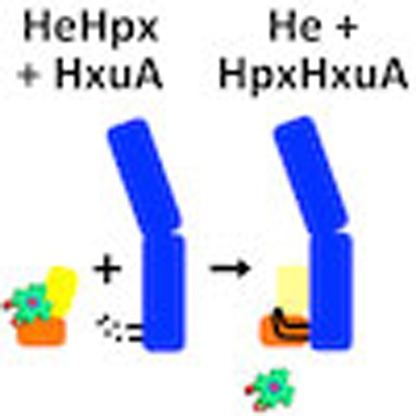

Haem-binding proteins, which contain >75% of the iron in higher organisms, are the major sources of iron for all commensal/pathogenic organisms that develop in mammals. Haem uptake from host haemoproteins requires a recognition step, and an internalization step. Internalization across the cell membranes is usually the only step that requires energy. Energy is provided by proton-motive-force dissipation (TonB-dependent receptors) to cross the outer membrane of Gram-negative bacteria^1^ and ATP hydrolysis (in most cases, via ATPase subunits associated with cytoplasmic membrane bound permeases) to cross the cytoplasmic membrane of both Gram-positive and Gram-negative bacteria^2^,^3^. Haem can then be used as such or degraded via various pathways to retrieve the iron atom.

*Haemophilus influenzae* is a Gram-negative bacterium uniquely found in humans that asymptomatically colonizes the nasopharynx of up to 40% of the population. On crossing the epithelium barrier, it can cause several diseases, from otitis to pneumoniae and meningitis, mostly in young children and immunocompromised patients. Besides its iron requirement, *H. influenzae* also displays an absolute haem prerequisite to sustain its aerobic growth, as it is devoid of most haem biosynthesis enzymes^4^. For this reason it has developed several systems that allow the acquisition of haem from host haemoproteins, such as haemoglobin, haemoglobin–haptoglobin and haemopexin^5^,^6^. The haem-haemopexin acquisition system is particularly interesting because *Haemophilus influenzae* is one of the few bacterial species that can use this haemoprotein as a source of haem and iron.

Haemopexin is a 50-kDa serum glycoprotein involved in haem recycling after haemolysis. It has one of the highest known affinities for haem (Kd ca. 0.1 pM), and is present in all mammalian body fluids including cerebrospinal fluid^7^. Its concentration (in normal conditions ∼10 μM in serum) increases on inflammation. The X-ray structure of rabbit haem-haemopexin has been solved (PDB codes 1QHU and 1QJS). It is made up of two homologous halves (four-bladed β-propeller architecture, defining the matrix metalloproteinase MMP fold) connected by a ca. 20 residues linker. Haem is located at the boundary of the two MMP domains, two histidine residues (one from the linker, one from the C-terminal domain) acting as iron axial ligands^8^.

The system that allows haem uptake from haemopexin (Hxu system), comprises three proteins: HxuB and HxuA, which together form a two-partner-secretion (Tps) system^9^,^10^,^11^, and the TonB-dependent haem receptor HxuC. In invasive *H. influenzae* strains, HxuA is exposed at the cell surface. It belongs to the family of TpsA cargo proteins that are secreted through their specific TpsB partner. TpsB's are members of a superfamily of 16-stranded β-barrel proteins involved in protein secretion across, and protein incorporation into, the outer membrane. TpsA proteins are cell surface/secreted virulence factors, and can be divided into at least two subfamilies: the filamentous haemagglutinin adhesin (FHA) subfamily, and the high-molecular-weight adhesin (HMW) subfamily (to which HxuA belongs). The HMW subfamily is defined by the presence of a disulfide bridge at the C-terminus, involved in anchoring the TpsA at the cell surface. In both subfamilies the highly conserved N-terminal Tps domain is involved in the recognition/secretion by the TpsB partner^11^. Previous studies showed that HxuA makes high-affinity stoichiometric complexes with haemopexin or haem-haemopexin and that haem is released from haem-haemopexin on interaction with HxuA, becoming available for the receptor HxuC (ref. 12). To our knowledge, no full-length structure of a TpsA protein is available. However, *in silico* models predict the fold of these proteins as long β-helices^11^. The only structures reported so far are of Tps domains from several TpsA proteins^13^,^14^,^15^,^16^.

There are few systems where capture of haem from host haemoproteins by the bacterium is understood at the molecular level, and the Hxu system appears to be well-suited to tackle the open questions. Here we report the crystal structures of full-length HxuA and HxuA in complex with the N-terminal domain of rabbit haemopexin, together with HxuA mutants characterization, both *in vivo* and *in vitro*. We propose a detailed molecular mechanism for haem acquisition from haemopexin.

## Results

### Crystal structure of HxuA

**Figure 1:**
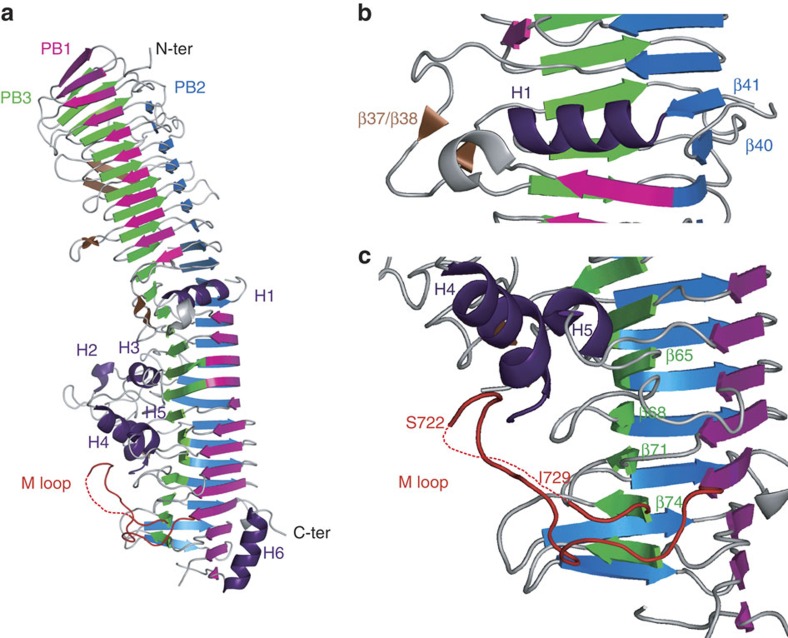
X-ray structure of the HxuA molecule. (**a**) Cartoon representation of HxuA, a right-handed β-helix. Parallel β-sheet PB1 is coloured in magenta, PB2 in blue, PB3 in green and the extra-helix strand motifs in brown. The α-helix elements H1–H6 are shown in purple. (**b**) Enlarged view of α-helix H1, which splits PB1 into two parts. Together with the extra motifs β37/β38 and the swap of β40 and β41, this element induces a twist in the β-helix, highlighting the separation between the secretion domain and the functional domain. (**c**) Enlarged view of loop 706–731, in red, referred to as the M loop, which undergoes an important conformational change during complex formation.

HxuA (884 residues) contains two cysteine residues close to the C-terminal end, at positions 855 and 861. These two residues are thought to form a disulfide bridge, which likely anchors the protein in its TpsB translocator^17^. A variant of HxuA was engineered, in which these two cysteines were replaced by serines, allowing more efficient release of the protein into the supernatant^12^. The X-ray structure of this variant (referred to as HxuA throughout) was solved at 1.6 Å resolution, and is presented in Fig. 1a together with the crystallographic data (Table 1, Supplementary Fig. 1a). HxuA is an elongated molecule that folds into a ca. 120 × 35 × 35 Å kinked right-handed β-helix; it is made up of two distinct parts of roughly equal length. The N-terminal domain (residues 1–350, encompassing the TPS domain^14^) is characteristic of this family of proteins, and referred to as the secretion domain. The C-terminal domain (residues 351–884) is by anticipation referred to as the functional domain. C-terminal residues 818–884 were not seen in the electron density map. However, mass spectrometry analysis of a dissolved crystal indicated that the protein was largely intact in the crystal (Supplementary Fig. 2a, bottom, as compared with Supplementary Fig. 2a, top). The crystal structure of the Tps secretion domain of HxuA has been reported previously^14^. Except for crystal contacts, there do not appear to be significant differences, between this domain in its isolated form and in the full-length protein.

The resolution of the structure of full-length HxuA reveals that the protein is fully organized as a right-handed β-helix, with three parallel β-sheets PB1, PB2 and PB3 (Supplementary Table 1 for the definition of secondary structures). PB2 and PB3 each have 24 strands and are continuous, while PB1 is split into two parts, one part containing 11 strands (towards the N-terminus) and the other 13 strands (towards the C-terminus). These two parts are separated by a 10 residue-long α-helix (H1) and a 21 residue-long insertion between strands β36 and β39. This insertion contains two short β-strands β37/β38 (β37(Tyr314-Asn316)/β38(Arg320-Asp322), Supplementary Table 1) that lie nearly perpendicular to the axis of the C-terminal part of the β-helix. At the junction between the secretion and the functional domains, strands β40 and β41 are also swapped within PB2 (Fig. 1b). Together these features induce a kink in the relative orientations of the secretion and functional domains and a twist in the β-helix itself, which might ensure a strong physical link between the two parts as well as some plasticity at the junction of the two domains. The C-terminal functional domain contains very long insertions between the strands of the parallel β-sheets. These insertions cover one face of the β-helix formed by PB3. All of the insertions or loops are thus folded onto the floor of a single parallel β-sheet, giving the C-terminal region of the molecule a highly asymmetric shape. The longest insertion (residues 512–578), located between strands β57 (in PB3) and β60 (in PB2) is folded onto most of the C-terminus, and contains additional secondary structure including helices H3, H4 and H5 (Fig. 1a,c). This loop also contains two short additional distorted β-strands (β58/β59). Several segments within loops are not seen in the electron density map and, given the overall resolution of the structure, likely correspond to mobile portions of the molecule. One loop, hereafter referred to as the M (for moving) loop (residues 706–731, between strands β73 and β74 in, respectively, PB1 and PB3), which we showed to play an essential role in haem release from haemopexin (see below) is not completely defined in the electron density map (residues 723–728 are missing). The M loop conformation is stabilized by several H-bonds/salt bridges (Supplementary Table 2) involving residues from the 713–722 segment locking the loop in a groove delineated by strands β65, β68, β71, β74, helices H4 and H5 and the β64-β65 loop (Fig. 1c). Finally, a C-terminal 12 residue-long amphipathic α-helix (H6, residues 802–814) is folded back onto PB1 and faces strands β73, β76, β79 and β80, with its polar residues oriented towards the exterior of the molecule. Hydrophobic interactions with strands from PB1 stabilize its conformation.

### HxuA only interacts with the N-terminal domain of haemopexin

The resolution of the HxuA structure led us to propose that the interaction surface of HxuA with haemopexin is probably provided by the long insertions and structural motifs folded on PB3 of the C-terminal part of the β-helix. However, it gave no clues about how this could happen and how haem is released during the interaction of HxuA with haem-haemopexin. Haemopexin is made of two structurally similar, four-bladed β-propeller MMP domains (residues 1–208 and 228–435) connected by a flexible interdomain linker that contains one of the two haem iron ligands (histidine 213 in the case of rabbit haemopexin), the other one (His 266) being in the C-terminal domain^8^. It was reported previously that the two propeller domains can be separated by mild proteolytic digestion^18^. The isolated N-terminal domain (residues 1–208) still has some affinity for haem, and both domains have affinity for each other that is reinforced in the presence of haem^18^. It was, therefore, relevant to investigate whether HxuA had affinity for both haemopexin propeller domains or just one. To this end, we purified both domains separately, and tested their putative association with HxuA by isothermal titration calorimetry (ITC) and size exclusion chromatography (SEC).

**Figure 2:**
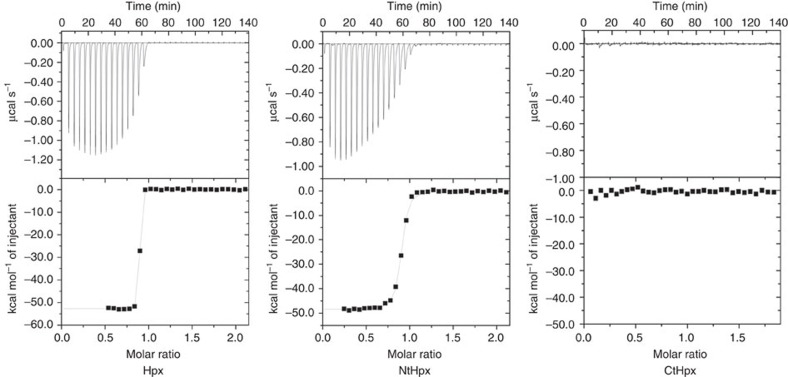
Interaction of HxuA with haemopexin and its domains. Interaction of HxuA with haemopexin (Hpx) and the N and C-terminal fragments of haemopexin (NtHpx and CtHpx, respectively), was measured by ITC. The upper part shows the heat signal for the titration. The lower part of each panel shows the binding isotherm derived from the heat signal, together with the fit calculated with Origin software.

We previously demonstrated that HxuA forms high-affinity stoichiometric complexes with haemopexin (ΔH=−47.3 kcal mol^−1^, Ka>10^9 ^M^−1^) (ref. 12). Figure 2 shows the thermograms of the interaction of HxuA with isolated domains of haemopexin, and with full-length haemopexin as a control. No heat exchange signal was detected with the C-terminal haemopexin domain (CtHpx), but a very strong signal was observed with the N-terminal haemopexin domain (NtHpx). Data analysis showed that a ca. 1:1 complex was formed in the latter case, and that the heat evolved during the interaction was almost equal to that observed with the full-length protein (−48.5 versus −52.7 kcal mol^−1^ in this particular experiment). This indicates that NtHpx likely provides most, if not all, of the bonds responsible for the interaction with HxuA, with little or no contribution from the C-terminal domain. The affinity constant measured for NtHpx is smaller than that determined for the full-length protein (Supplementary Table 3). The results were confirmed by SEC; NtHpx formed complexes with HxuA but CtHpx did not (see Supplementary Fig. 2b,d,e).

### Transmission Electron Microscopy Negative Stain

We used negative stain transmission electron microscopy (TEM) to obtain additional topological information about the interactions between HxuA and haemopexin. In agreement with the X-ray data, HxuA was shown to have a ca. 140 Å long and, on average, a 50 Å wide rod-like structure (Supplementary Fig. 3a). At this resolution, the asymmetric shape of one end attributable to the C-terminal β-helix insertions, is not obvious. By contrast, for haemopexin two domains corresponding to the two MMP domains were clearly identified (Supplementary Fig. 3b).

**Figure 3:**
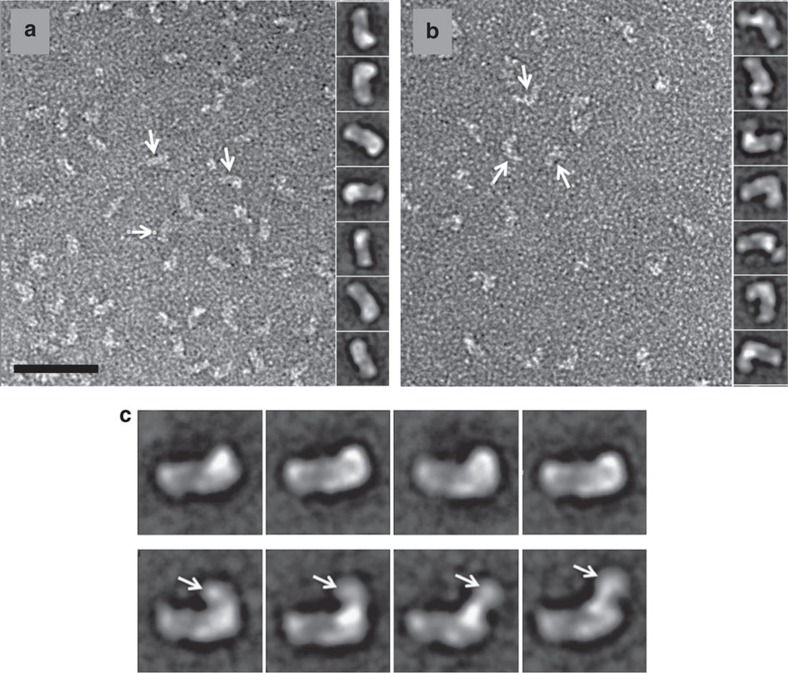
TEM analysis of the HxuA-NtHpx and HxuA-Hpx complexes. (**a**) Micrograph of the negatively stained HxuA-NtHpx complex. The arrows indicate the protein particles adsorbed on the carbon film. The right panel shows a representative gallery of class averages from 7,104 particle projections analysed using the EMAN2 software. (**b**) Micrograph of the negatively stained HxuA-Hpx complex. The arrows indicate the protein particles adsorbed on the carbon film. The right panel shows a representative gallery of class averages from 9,847 particle projections analysed using the EMAN2 software. In **c**, the arrow indicates the extra density clearly visible on the side views of the HxuA-Hpx complex (bottom) compared with the HxuA-NtHpx complex (top panel). The scale bar in **a** is 50 nm. The boxes are 18 nm in **a** and **b** and 20 nm in **c**.

Binding of NtHpx to HxuA resulted in extra density at one end of the elongated HxuA molecule, while the other end appeared more regular, and might thus be the N-terminal part of the β-helix (Fig. 3a). The complex formed by HxuA with full-length haemopexin (HxuA-Hpx), had even more density at the same HxuA end (Fig. 3b). We attribute this additional density to the C-terminal domain of Hpx. Notably, the extra density does not always occupy the same position with respect to the rest of the molecule, but is found in a limited number of preferential positions, as documented by the class averages (Fig. 3c). These results suggest that, most likely due to the flexible linker between the two MMP domains, the C-terminal domain of Hpx does not occupy a fixed position in the HxuA-Hpx complex.

### X-ray structure of the HxuA-NtHpx complex

**Figure 4:**
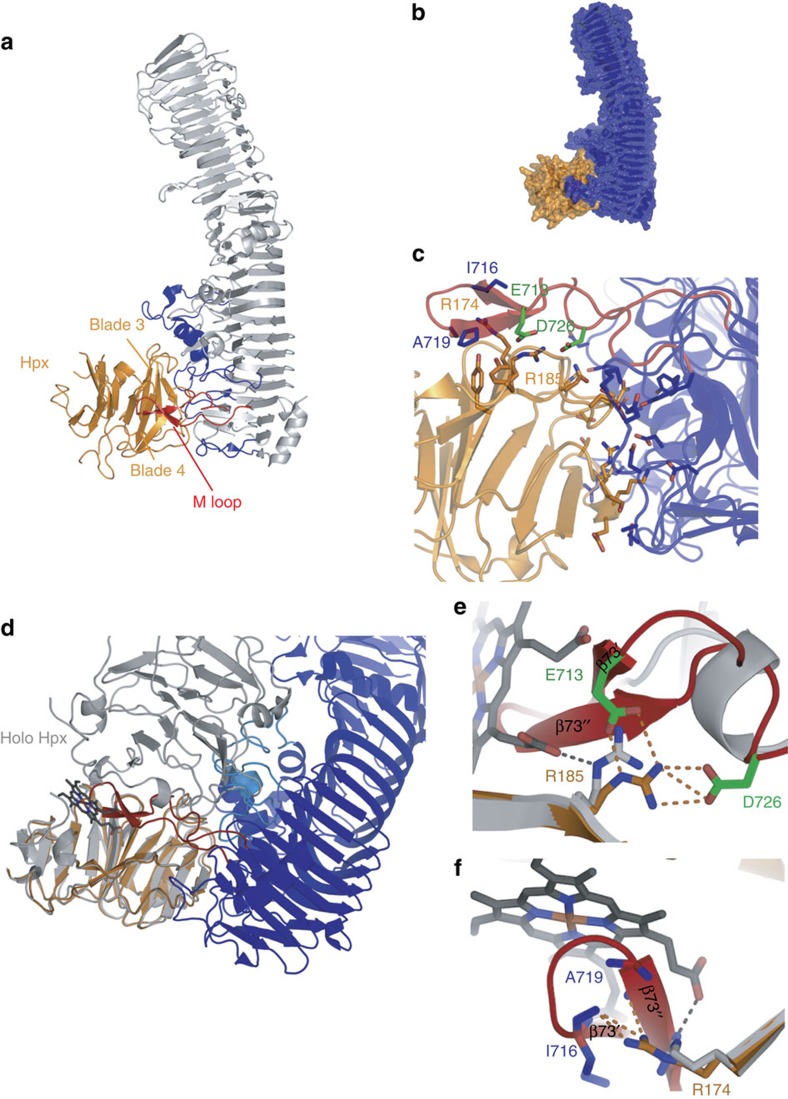
X-ray structure of the HxuA-NtHpx complex. (**a**) Cartoon representation of HxuA (silver) in complex with the N-terminal domain of haemopexin (NtHpx; orange). The secondary structure elements in HxuA that provide residues for the interaction with residues from blades 3 and 4 of NtHpx are coloured blue. The M loop is coloured red. (**b**) Surface representation of the complex between HxuA (blue) and NtHpx (orange). (**c**) Enlarged view of the interaction zone. The side chains of residues involved in hydrogen bonds or salt bridges formation is represented in sticks and coloured as in 4 A side chains of residues Glu713 and Asp726 are shown in green. (**d**) Superposition of full-length haem-haemopexin (1QHU, in grey) with the structure of the HxuA-NtHpx complex (in blue and orange), showing the potential steric clashes of the C-terminal domain of haemopexin with HxuA (pale blue) and of haem with the M loop of HxuA (red). (**e**,**f**) Enlarged view of the region of interaction involving the M loop together with the reorientation of side chains of Arg174 and Arg185 from haem-haemopexin. The dashes represent atoms located within hydrogen bonding distances.

All attempts to obtain crystals of full-length haemopexin in complex with HxuA failed, probably because the linker joining the two domains is flexible and the C-terminal domain occupies several positions. This, together with the demonstrated interaction of HxuA with the N-terminal domain of haemopexin (Section 2) led us to undertake the crystallization of the HxuA-NtHpx complex. This complex gave many hits on high-throughput crystallization screening, and its structure was solved at 2.8 Å resolution by molecular replacement with both our previous HxuA structure and the N-terminal domain of haem-haemopexin derived from the 1QHU structure^8^ as templates (Fig. 4a, Supplementary Fig. 1b and Table 2). NtHpx binds at the C-terminal end of HxuA. The overall shape of the HxuA molecule is well conserved between the free and bound states (rmsd =0.76 Å over 757 Cα atoms (773 total), excluding residues 712–723 from the M loop, Ser765-Lys766 from another C-terminal loop making contacts with haemopexin, and residues Asn221 and Ala223 from another loop in the N-terminal part), as is the shape of the N-terminal domain of haemopexin (rmsd=0.58 Å over all Cα atoms). The N-terminal domain of haemopexin folds as a four-bladed β-propeller, with each propeller blade comprised of a three- or four-stranded, antiparallel β-sheet, the first and fourth blades being tied together by a disulphide bond between Cys27 and Cys208 (ref. 8). The respective orientation of the two molecules in the complex is such that the fourfold symmetry axis of the NtHpx four-bladed β-propeller is almost parallel to the axis of the HxuA β-helix (angle of ca. 20°). The surface of interaction (Fig. 4b) buries ∼1600 Å^2^ and the contact zone is highly polar (Supplementary Table 4 and Fig. 4c). The region between residues 706 and 731, which we named the M (moving) loop, is of particular importance. This loop undergoes a very large movement (26 Å for example for Cα of Gly718) on haemopexin binding. In HxuA alone, the M loop is locked in a groove delineated by strands β65, β68, β71, β74, helices H4 and H5, and the β64-β65 loop. The folding of two short β-strands between residues Glu713-Ile716 (β73') and Ala719-Ser722 (β73''; see, for example, Fig. 4d–f) of the M loop is induced by haemopexin binding, and occurs concomitant to the large conformational change. These β-strands contribute many interactions with blade 4 of haemopexin (Supplementary Table 4). In haemopexin, the interaction with HxuA only involves residues from blades 3 and 4 (Fig. 4a). First, within blade 3, residues located between strands 1 and 2, between strands 3 and 4, and within strand 4 interact with helix H4 and loops β51-β52, β57-β60, β67-β68, β71-72 and β74-β75. Second, within blade 4, residues located in strands 2 and 3 and also in the insertion between strands 3 and 4 interact with residues from strands β71, β73', β73'' and loops between strands β73''-β74 and β79-β80. All polar interactions are listed in Supplementary Table 4. Thus, the surface of interaction can roughly be divided into two patches, one coming from the moving M loop, and the other from regions of the β-helix that undergo much less extensive rearrangement on haemopexin binding and are set free on M loop movement (Fig. 4c).

Close examination of the structure of the HxuA-NtHpx complex also provides a rational basis for haem release on interaction of haem-haemopexin with HxuA. Superposition of the complete haem-haemopexin structure on the NtHpx in the complex indicates that the C-terminal domain of haemopexin would sterically clash with HxuA, mostly with loops β57-β60, β64-β65 and β67-β68 of HxuA that are folded over the β-helix, as well as with the M loop (Fig. 4d). Furthermore, analysis of the M loop contacts with NtHpx also indicates that there would be a direct steric clash between Val-Ile714-715, Gly-Ala-Pro718-720 and both haem propionates in the haem-binding site in haemopexin (Fig. 4d). Indeed, residues of the M loop make polar bonds with residues from haemopexin involved in haem binding and distort its binding pocket. In particular, in haem-haemopexin, residues Arg174 and 185 (blade 4; strands 2 and 3 respectively) make polar bonds with one of the propionates of the haem molecule. In the complex, their side chain orientation is modified, further weakening haem binding to Hpx (Fig. 4e,f). Moreover Arg185 from haemopexin makes polar bonds with side chains of residues Asp726 (loop β73''-β74) and Glu713 (β73''), while Hpx Arg174 is further stabilized by interactions with the carbonyl groups of Ile716 and Ala719 within HxuA (Supplementary Table 4). We therefore hypothesized that the whole-M-loop and in particular some of its residues would play an essential role in haem ejection from haem-haemopexin.

### Key role of the M loop in haem ejection from haem-haemopexin

**Figure 5:**
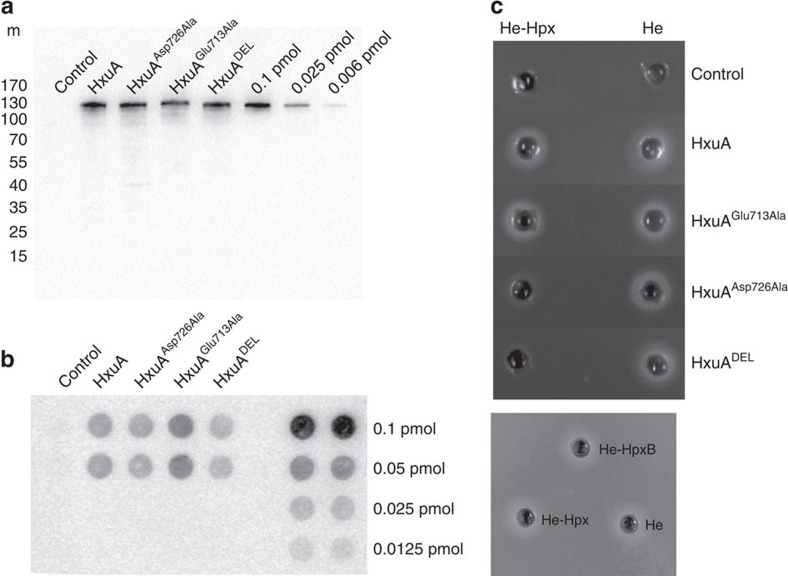
Phenotypes of HxuA mutants. (**a**) Amount of HxuA in the various strains, as detected by immunoblotting with anti-HxuA antibodies on whole-cell contents analysed by SDS-PAGE (control, WTHxuA, HxuA^Asp726Ala^, HxuA^Glu713Ala^ and HxuA^DEL^). The equivalent of 0.05 OD_600nm_ (10^7^ cells) was deposited in each lane. Known amounts of purified HxuA were run alongside. The scale on the left (m) represents molecular weight in kDa. (**b**) Detection of interaction of biotinylated haemopexin with HxuA and its mutants in whole cells by dot-blot analysis *in vivo* (control, wild-type, HxuA^Asp726Ala^, HxuA^Glu713Ala^, HxuA^DEL^). Samples were spotted in duplicate. The equivalent of 0.05 OD_600nm_ (10^7^ cells) was deposited in each spot, in duplicate. Known amounts of purified biotinylated haemopexin were run alongside, in duplicate. (**c**) Analysis of the *in vivo* activities of HxuA, HxuA^DEL^, HxuA^Glu713Ala^ and HxuA^Asp726Ala^, in haem acquisition tests from haem and haem-haemopexin. Each well contained 100 μl of either 5 μM haem (He) or 5 μM haem-haemopexin (He-Hpx). The bottom panel shows the same activity test for haem (He), haem-haemopexin (He-Hpx) and the biotinylated haem-haemopexin (He-HpxB) for WT strain.

We constructed three HxuA mutants to test this hypothesis. Their phenotypes were first tested *in vivo* with respect to their production (Fig. 5a), their ability to bind haemopexin in a dot-blot assay on whole cells (Fig. 5b) and their ability to deliver haem from haem-haemopexin to the HxuC receptor in the reconstituted system in an *E. coli* haem auxotroph strain (Fig. 5c). The first, HxuA^DEL^, had a deletion of most of the M loop (residues 711–728, see MM for details). In the *hxuCBA* context reconstituted in *E. coli*, this mutant protein was correctly produced at ca. wild-type level (Fig. 5a), and was able to make complexes with haemopexin *in vivo*, as tested by dot-blot on whole cells using biotinylated haemopexin (Fig. 5b). It did not allow haem acquisition from haemopexin, but had no effect on free haem acquisition (Fig. 5c). The two other mutants were point mutants in Asp726 and Glu713, two residues that establish H-bonds/salt bridges with Arg185 from the N-lobe of haemopexin (one of the residues that stabilize haem propionates in haem-haemopexin) (Fig. 4e). Furthermore, the Arg185 side chain reorientation on interaction with HxuA is most likely driven by Asp726. Glu713 and Asp726 were replaced by alanine, generating HxuA^Glu713Ala^ and HxuA^Asp726Ala^ mutants. The HxuA^Glu713Ala^ mutant behaved the same as the wild-type in the various tests (Fig. 5a–c), and was not studied further. This was not the case for HxuA^Asp726Ala^ mutant (produced at wild-type level, Fig. 5a), which was strongly affected in its ability to allow growth on haem-haemopexin (Fig. 5c) but able to bind haemopexin *in vivo* (Fig. 5b). Compared with the M loop deletion mutant, this mutant displayed barely visible, but reproducible, ability to acquire haem from haem-haemopexin (Fig. 5c).

To gain more information on the interaction of those two mutants (HxuA^DEL^ and HxuA^Asp726Ala^), the cysteine-less variants of these mutants were constructed, the corresponding proteins were purified from the supernatant and their interaction with haem-haemopexin tested by both ITC and SEC experiments. ITC experiment (Supplementary Fig. 4) indicated that HxuA^DEL^ interacted with haem-haemopexin but with different parameters compared with the WT (Supplementary Table 3); a 1:1 complex was formed even though the ΔH was substantially lower (−13.0 kcal mol^−1^ versus −39.8 kcal mol^−1^), and the Ka was also affected (1.7 × 10^8^ M^−1^ versus>10^9^ M^−1^). The formation of the complex was also confirmed in SEC experiments (Supplementary Fig. 5) and the complex strikingly contained haem, as evidenced by the absorbance at 410 nm. The absorption spectrum of the complex was very similar to that of haem-haemopexin in the visible part of the spectrum, suggesting that haem did not drastically change environment on interaction (Supplementary Fig. 5b,c). These results pinpoint an essential role for the M loop in haem ejection from haemopexin, since the interaction with haemopexin takes place without the M loop, but haem ejection is affected. Similar experiments were carried out with the cysteine-less variant of the HxuA^Asp726Ala^ mutant. The mutant protein also formed stoichiometric complexes with haem-haemopexin (Supplementary Fig. 4). Both ΔH and Ka were affected compared with the wild type (Supplementary Table 3) (−12.8 kcal mol^−1^ versus −39.8 kcal mol^−1^, and 2.9 × 10^6^ M^−1^ versus >10^9^ M^−1^, respectively). After gel filtration, the purified complex still showed absorbance in the Soret region, indicating that haem was present, and the absorption spectrum was not significantly different from that of haem-haemopexin, except for a 2-nm red-shift of the Soret band maybe indicative of a slight distorsion of the haem geometry on interaction (Supplementary Fig. 5b,c). There was a detectable reduction in the ratio of the 414 nm/280 nm absorbance as compared with the HxuA^DEL^-haem-haemopexin complex, potentially indicating partial release of haem on complex formation (Supplementary Fig. 5), which could explain the minute but detectable growth observed with this mutant. These results indicate that a single residue, Asp726 (that is conserved in all HxuAs^19^), is essential for haem acquisition from haem-haemopexin. Finally, whereas the ΔH's of interaction of the two HxuA mutants with haem-haemopexin are quite similar (Supplementary Table 3), they are quite different with haemopexin (Supplementary Table 3). This might indicate that, as there is almost no haem release from haem-haemopexin by the HxuA^Asp726Ala^ mutant, the polar interactions between haem-haemopexin and HxuA mutants are similar. On the other hand, with haemopexin, the M loop from the HxuA^Asp726Ala^ mutant could establish close to wild-type contacts with haemopexin, which obviously could not form with the HxuA^DEL^ mutant.

It is therefore likely that, at least, a two-step process occurs. Initially, haemopexin would be captured by HxuA, without gross modification of the haemopexin structure and haem ligation; this might correspond to the state stabilized by the Asp726 mutant. Steric clashes between the C-terminal domain of haemopexin and HxuA together with the insertion of the M loop in the cleft between the two domains, up to the interaction of Asp726 from HxuA M loop with specific haemopexin residues, would then trigger haem release and its capture by the HxuC receptor.

## Discussion

Haem acquisition is a major route by which pathogens/commensals take up iron. In most cases, it depends on bacterial haemophores that recognize either haem and/or the host haemoprotein^20^. The purpose of haemophores is twofold, first to allow a very efficient haem/haemoprotein capture, second to ultimately trigger haem transfer to a membrane bound component that actively transports haem across the bacterial membrane. Two haemophores classes have been particularly well studied, the HasA (Gram-negative) and NEAT (Gram-positive) types. In both cases, interaction with haem and haem transfer to the membrane bound transporter have been extensively studied^21^,^22^,^23^, but the precise mechanism of interaction with the host haemoprotein leading to haem extraction are not understood. HxuA defines a new class of haemophores that promote haem release from haemopexin without having a detectable affinity for haem^12^.

HxuA belongs to the same class of TpsA molecules as HMW1A. It folds as a right-handed β-helix, as expected for a TpsA protein^24^. The N-terminal secretion domain terminates with a α-helix not predicted from the primary sequence. Together with a strand swapping in one of the parallel β-sheets (β40-β41), this helix introduces a small bend and a change of register in the whole molecule. Whether this is a conserved feature of TpsA molecules or specific to HxuA remains to be determined. FHA and HMW1A display extended signal peptides characteristic of the TPS pathway. This is not the case for HxuA, maybe indicating differences in the secretion mechanism. The structure itself does not allow further conclusions.

The various TpsA proteins function in different ways. They range from adhesins (FHA from *B. pertussis*, HMW1A from *H. influenzae*)^25^,^26^,^27^ to pore-forming toxins^28^,^29^,^30^ to proteases^31^. HxuA defines a specific class of haemophores. Its function requires the precise binding of haemopexin so as to release its bound substrate (haem). This is ensured by the very long insertions, which protrude from the β helical scaffold in the C-terminal part and form the haemopexin binding site.

The last 69 residues of HxuA are not seen in the X-ray structure. Yet, the mass spectrometry data indicate that this part of the molecule is not cleaved, at least for a substantial proportion of the molecules, indicating that this C-terminal part is likely disordered. Like HMW1A, HxuA has two cysteine residues at the C-terminus. In HMW1A, these somehow anchor the molecule to the outer membrane. It is believed that, like in the HMW1A case, the extreme C-terminus of HxuA with this disulfide bridge resides in the periplasm (residues 854–884) and that the preceding sequence (residues 824–853) resides in the HxuB barrel as an α-helix. FhaC, the TpsB of the FHA system, can accomodate an α-helix in its barrel^32^. It is therefore likely that a very short linker (residues 819–823) connects the end of our structure (residue 818) and the helix inserted in the HxuB barrel, putting the HxuA molecule very close to the cell surface.

A plausible scenario for haem acquisition would be as follows: HxuA lies on the cell surface with its C-terminal amphipatic helix directed towards the outer membrane, and the protruding loops from the C-terminal domain directed towards the extracellular milieu. Haemopexin capture takes place at the C-terminal end of HxuA by a highly polar/charged interface. Rearrangement of the M loop in the cleft between the two MMP domains of haemopexin, could trigger additional conformational changes in haemopexin, weakening haem binding and leading to haem release and its capture by the HxuC receptor. Several points support this model. First, there is one highly negatively charged patch at the HxuA surface (Asp563, Asp685, Asp738 from different loops at the HxuA surface) that comes in close contact with a positive patch on Hpx (Lys155, Arg157). Second, M loop movement from its initial position to its position in the complex, might also be driven by ionic interactions between the positively charged haemopexin and the negatively charged M loop. This is coherent with the very different interactions measured between haemopexin and wild-type HxuA on the one hand, and between haemopexin and HxuA^DEL^ on the other hand. This is also coherent with the similar interactions measured between haemopexin and wild-type HxuA on the one hand, and between haemopexin and HxuA^Asp726Ala^ on the other hand. The water molecules that lie at the interface of the two haemopexin domains have been envisioned as key players in relative movements of both domains leading to haem release^8^, and it is obvious that M loop positioning at the contact of the N-domain of haemopexin will also lead to water molecules displacement. In the hepatocyte, although there is debate about the identification of the genuine haemopexin receptor^33^,^34^, receptor driven endocytosis leads to haem-haemopexin internalization and haem release probably occurs in endocytic vacuoles at a pH below 5, likely associated with histidine protonation. This is not the case for haem release from haem-haemopexin by HxuA, which leads to question the relative contribution of the different elements to haem binding, respectively histidine residues, hydrophobic stacking to the pyrrole ring from several residues and propionate stabilization by positively charged residues.

Regarding the late steps of this process, it is possible that the HxuC receptor might interact with either HxuA or the HxuA-haemopexin complex to efficiently capture haem released from haemopexin. We have previously shown that HxuAdm (the cysteine-less variant of HxuA) is inactive in the context of the reconstituted system in *E. coli,* despite being perfectly able to discharge haem from haem-haemopexin *in vitro*. Our current hypothesis is that function of the Hxu system requires dissociation of haemopexin from the HxuA molecule, once haem from haem-haemopexin has been taken up by the HxuC receptor. This dissociation step would require energy transduced by the TonB complex, and hence a direct interaction with either TonB or HxuC is likely to exist.

In contrast to the *N. meningitidis* TbpA/TbpB receptor, which is exquisitely specific for human transferrin^35^, the Hxu system from *H. influenzae* can accept haemopexins at least from human and rabbit^36^. When the structures of human and rabbit haemopexin (82% sequence identity) were compared, all haemopexin residues interacting with HxuA residues by their side chains are conserved, with one exception. Also, for TbpA/TbpB-transferrin, it is very likely that, TbpB fulfills a shielding function to avoid Fe leakage towards the extracellular medium as well as discriminating towards Fe-transferrin. In the HasA-HasR-haem case, haem still has access to its binding site on HasR from the extracellular medium^22^,^37^,^38^. This is not yet known for the HxuA-haemopexin-HxuC system. In both the HasA-HasR and TbpA-TbpB-transferrin systems, haem/iron release is carried out by extracellular loops of the TonB-dependent receptor^22^,^39^, which in turn releases the discharged carrier protein. This is clearly different from the Hxu system, where haem release from haemopexin does not involve the TonB-dependent HxuC receptor. As compared with the strategies used by Gram-positive bacteria, examplified by the Isd system from *S. aureus* where the initial capture of haem from haemoglobin is insured^40^ by NEAT domains containing IsdB or IsdH. In both cases, a NEAT domain having no affinity for haem but for haemoglobin, is linked via a linker domain that allows the correct positioning of a second NEAT domain having affinity for haem, and very little affinity for haemoglobin. Haem is then transferred through several NEAT domains by a so-called hand-clasp mechanism^23^ to the permease.

Finally, it is also possible that HxuA, besides its function in preventing haem binding to haemopexin, also prevents haemopexin binding to its specific hepatocyte receptor, as it partially covers an epitope (residues 120–142), essential for this interaction^41^.

## Methods

### Protein expression and purification

*Full-length HxuA*. HxuA was expressed in *E. coli* BL21 transformed with a pBAD24 derivative harbouring the *hxuCBA* operon. The two last cysteine codons of *hxuA* were respectively changed to serine, to obtain a higher release of HxuA into the culture supernatant^12^.

The strain was grown in a 4-l fermentor at the Institut Pasteur Protein Production Facility, in medium density medium, with 80% pO2 throughout at 37 °C. At an OD_600 nm_ of ca. 4–5, the culture was induced with arabinose (20 μg ml^−1^) for ∼150 min, up to a maximum OD_600nm_ of 30. After quick cooling to ca. 10 °C, the culture was centrifuged (20 min at 10,000*g*). The supernatant was withdrawn and quickly supplemented with EDTA (10 mM final), Tris base (1 mM final) and ammonium sulfate (80% saturation). After incubation at 4 °C for 1 h, the supernatant was centrifuged (45 min at 12,000*g*). The resulting pellet was resuspended in 20 mM Tris-HCl pH 7.5, 150 mM NaCl, 10 mM EDTA and dialyzed against the same buffer overnight. The supernatant was centrifuged for 3 h (at 200,000*g*) at 4 °C, to eliminate insoluble material, filtered through a 0.2-μm filter and concentrated using an Amicon-Ultra 15 Ultracel 50 K (50 kDa cutoff) device down to a volume of ca. 30 ml. The retentate was filtered through a 0.2-μm filter and a SEC step (Superdex 200 pg 26 × 60) was run in the same buffer and 4 ml fractions collected. Fractions of interest (as analysed by SDS–polyacrylamide gel electrophoresis (SDS-PAGE)) were pooled, concentrated down to <1 ml, then the buffer was exchanged for 20 mM Tris-HCl pH 9.0, 15 mM NaCl, 1 mM EDTA and finally loaded on a monoQ HR 10–100 column. Sample was eluted with a NaCl gradient from 15 to 500 mM in the same buffer over 15 column volumes. Fractions (0.5 ml) were collected, analysed by SDS-PAGE and pure fractions of HxuA were pooled, concentrated up to 35–40 mg ml^−1^ for immediate crystallization trials, or kept frozen at −80 °C until use. The same protocol was employed for HxuA mutants. HxuA concentration was estimated after amino acid analysis of a representative preparation at the Unité de Chimie des Biomolécules (Institut Pasteur). The amino acid composition was in excellent agreement with HxuA composition and led to an estimated molar extinction coefficient of 50,990 M^−1^ cm^−1^ instead of the calculated one of 44,500 M^−1^ cm^−1^. The same sample analysed by mass spectrometry (Supplementary Fig. 2) gave a mass of 96,467.6094 (theoretical mass: 96,469.6926).

*Rabbit haemopexin*. Hpx was purified from 500 ml of rabbit serum (Biosera, minimal haemolysis), by a modification of the protocol described by Morgan^41^. Briefly, phenyl methyl sulfonyl fluoride (at 0.1 mM final) was added to thawed rabbit serum. 62.5 ml of 1.8 M perchloric acid was slowly added under agitation at 4 °C, and agitation was continued for a further 10 min. The solution was centrifuged (20 min at 10,000*g*) and the supernatant was adjusted to pH 8.0 with 10 N NaOH. 50% (w/v) PEG 3400 (Polyscience) was added to a final concentration of 15%. After stirring for 1 h at 4 °C and centrifugation (20 min at 10,000*g*), the supernatant was adjusted with solid PEG 3400 to a final concentration of 25% (w/v), and stirred 1 h at 4 °C. After centrifugation (20 min at 10,000*g*), the pellet was resuspended in 20 mM Tris-HCl pH 8.0, concentrated using a Millipore centrifugal device (Amicon-Ultra 15 Ultracel 10 K), washed twice with the same buffer and finally with 20 mM MES-NaOH pH 6.0. The sample was loaded on a 20-ml S-sepharose column equilibrated with 20 mM MES-NaOH pH 6.0, and eluted with a linear gradient of NaCl from 0 to 1 M in the same buffer over 20 column volumes. Four millilitre fractions were collected and haemopexin eluted at ∼0.2 M NaCl. The fractions of interest were concentrated as above and the buffer was exchanged for 20 mM Tris-HCl pH 7.5. The sample was then loaded onto a mono Q HR10–100 column equilibrated with 20 mM Tris-HCl pH 7.5, and eluted with a linear NaCl gradient up to 1 M in the same buffer over 15 column volumes. The fractions (4 ml) were collected, pooled and then concentrated before being run on a SEC column (Superdex 200 pg, 26 × 60), which was equilibrated in 20 mM Tris-HCl pH 7.5, 150 mM NaCl and 10 mM EDTA. Four millilitre fractions of pure haemopexin, as judged from Coomassie blue staining and haem binding ability, were collected, pooled, concentrated and kept at -80 °C until use. The yield was ∼20 mg haemopexin starting from 500 ml of serum.

*N- and C-terminal sub-domains of haemopexin*. Haemopexin (1 ml at 30 μM, as estimated from the theoretical molar extinction coefficient of 118,090 M^−1^ cm^−1^ at 280 nm), was digested for 1 h at 37 °C with trypsin (0.2 μl, 1 mg ml^−1^). The reaction was stopped with phenyl methyl sulfonyl fluoride (1 mM final). Under these conditions the digestion was not complete, ∼1/3 of the initial haemopexin was still intact and two smaller fragments were observed. Those two smaller fragments, respectively, corresponding to the N-terminal and C-terminal domains of haemopexin were further purified by two successive steps of chromatography, first on a mono S HR10–100 column, then by SEC. N-terminal sequencing indicated that haemopexin was cleaved in the linker after Arg 214.

### Purification of the complexes

All complexes were purified by mixing a ca. 1.2-fold molar excess of either He-Hpx or the NtHpx fragment with either HxuA or its mutants, and running a SEC column (Superdex 200 pg, 26/60) equilibrated with 20 mM Tris-HCl pH 7.5, 150 mM NaCl, 10 mM EDTA.

### Crystallization

Initial screening of crystallization conditions was carried out for HxuA alone and for HxuA in complex with the N-terminal domain of rabbit haemopexin, using the vapour diffusion method with a Mosquito nanolitre-dispensing system (TTP Labtech). Sitting drops were set up using 400 nl of a 1:1 mixture of each sample protein and crystallization solutions (672 different commercially available conditions) equilibrated against a 150 μl reservoir in multiwell plates (Greiner Bio-One). The crystallization plates were stored at 18 °C in a RockImager1000 (Formulatrix) automated imaging system to monitor crystal growth. Manual optimization was performed in Limbro plates by the hanging-drop method. The best crystals of HxuA were obtained at a temperature of 18 °C by mixing an equal amount of protein solution (35 mg ml^−1^ HxuA) and reservoir solution (0.2 M MgCl_2_, 0.1 M HEPES, pH 7.5, 30% w/v PEG 400). Crystals appeared after 2–4 days. For the complex HxuA-NtHpx, crystals were obtained by the same technique using the protein solution at a concentration of ca. 50 mg ml^−1^ mixed with the following reservoir solution: 100 mM citric acid pH 5.60, 200 mM ammonium sulfate and 25% w/v PEG 4000. The time needed for crystals to appear was 1–2 weeks.

Single crystals of HxuA were flash-cooled in liquid nitrogen, using the mother crystallization liquid as cryoprotectant. For HxuA-NtHpx crystals, a mixture of 50% Paratone-N and 50% paraffin oil was used as cryoprotectant.

### Structure determination and refinement

X-ray diffraction data from single crystals (at −173 °C) for HxuA and HxuA-NtHpx were collected on beamline PROXIMA-1 at the Synchrotron SOLEIL (Saint-Aubin, France). Data were processed with XDS (ref. 42) and scaled with XSCALE, from the XDS package.

The structure of HxuA was solved by the single isomorphous replacement with anomalous signal method. A first data set was collected on a native crystal at a wavelength of *λ*=0.97918 Å. An Europium derivative was prepared by soaking a native crystal overnight in a solution containing Eu-(tris(pyridine-2,6-dicarboxylate) or DPA)_3_ lanthanide purchased from NatX-ray (Grenoble, France). A second data set was then collected on this derivative, at the energy corresponding to the peak of the Eu K-edge (48.5200005, keV, *λ*=1.775520 Å), as determined by an X-ray fluorescence scan. Two Eu sites were located using the programme SHELXD (ref. 43). Phasing was done with the programme Phaser^44^, and initial automatic model building proceeded with ARP/WARP (ref. 45). The model was then improved through iterative cycles of manual model adjustment and building with COOT (ref. 46). Final refinement was performed with autoBUSTER (ref. 47). Residues Ser1, Gly117-Gly121, Pro273, Lys274, Gly344-Thr349, Phe565-Gly571, Ile723-Asn728, Arg785-Lys793 and Ala818-Gln884 were not seen in the electron density map, and were not incorporated into the model.

The structure of HxuA in complex with the N-terminal domain of haemopexin was solved by molecular replacement with Phaser^44^ by using the structure of native HxuA and the N-terminal domain of haemopexin (PDB entry code 1QHU) as a template. Here also, the model was further improved through iterative cycles of manual model adjustment and building with COOT, and final refinement was performed with autoBUSTER. Residues Ser1, Ala2, Gly119-Gly121, Asp195, Asn196, Arg220, Pro273, Lys274, Val816-Gln884 from HxuA and residues Val1-Val23, Gly58-Leu62, Gly81, His82, Glu99-Val104 and Pro209-Arg214 from NtHpx were not seen in the electron density map and were not incorporated into the model.

All models were validated through the Molprobity server (http://molprobity.biochem.duke.edu)^48^. Figures were generated and rendered with Pymol (the PyMOL Molecular Graphics System, Version 1.5, Schrödinger, LLC.). The crystal parameters, data statistics and final refinement parameters are shown in Tables 1 and 2. Ramachandran statistics indicate that for HxuA 96.5% (742/769) of all residues are in favoured (98%) regions, and 100.0% (769/769) of all residues are in allowed (>99.8%) regions. Regarding HxuA in complex with N-ter haemopexin, 94.7% (908/959) of all residues are in favoured (98%) regions, and 99.7% (956/959) of all residues are in allowed (>99.8%) regions. Atomic coordinates and structure factors have been deposited in the Brookhaven Protein Data Bank under the accession numbers 4RM6 for HxuA and 4RT6 for HxuA in complex with N-ter haemopexin.

### Isothermal titration calorimetry

Proteins were dialyzed against 20 mM Na-phosphate buffer pH 7.0; the concentration of haemopexin and its fragments in the syringe was ∼100 μM and the HxuA concentration in the titration cell was ∼10 μM. ITC experiments were performed at 25 °C using the high precision VP-ITC system (MicroCal, GE Healthcare)^49^. In total, 35 individual 7 μl aliquots were injected from the 250 μl syringe into the 1.41 ml titration cell, with a 6-min interval between injections, unless otherwise stated. Heat signals were corrected for the respective heat of dilution and normalized to the amount of compound injected. The enthalpy of binding (ΔH), affinity constant (Ka) and molar binding stoichiometry (*n*) were directly obtained from the titration curve fitted using the single-site binding model of the Origin7software (OriginLab), even though there might be heterogeneity in the HxuA binding sites. HxuA concentrations were measured according to the experimentally determined molar extinction coefficient. Given the protein concentration in the titration cell (∼10 μM), the size of the injection and the limitation of the technique itself (Ka x substrate concentration between 1 and 1,000)^49^, the absolute value of the Ka determined should be taken with caution. Besides, as there are conformational changes in the interactants on interaction the values should be taken as apparent Ka's.

### TEM and image processing

For negative stain TEM, the protein solution was diluted at 10–20 μg ml^−1^ in PBS buffer. Immediately afterwards, 4 μl aliquot was adsorbed onto glow discharged carbon film coating copper EM grids, washed with three droplets of pure water and subsequently negatively stained with 2% (w/v) uranyl-acetate. The prepared grids were imaged using a Philips CM10 TEM (FEI Company, Eindhoven, the Netherlands) operating at 80 kV. The images were recorded by the 2k × 2k side-mounted Veleta CCD camera (Olympus, Germany) at a magnification of × 130,000. The pixel size at the sample level was 3.7 Å.

Image processing was carried out using the EMAN2 software package^50^. The images were contrast transfer function corrected and particle projections were semi-automatically selected. The e2refine2d programme was used to classify the particle projections. This programme produces reference-free class averages from a population of mixed, unaligned particle projections. The representative class averages with the best signal-to-noise ratio were selected and gathered in a gallery.

### Mutant construction

Plasmid phxuCBA was amplified with the following three oligonucleotides couples:

revglu 5′-CCA**GGCGCGCC**ATTAATGATCACTGCTTTCCCAGTTCTTCC-3′,

forglu 5′-AT**GGCGCGCC**TGGCTCAATTGATAATGATG-3′,

revasp 5′-CCA**GGCGCGCC**ATTAATGATCACTTCTTTCC-3′,

forasp 5′-AAT**GGCGCGCC**TGGCTCAATTGATAATGCTGCGAATATTGCC-3′,

revdel 5′-CCA**GGGCCC**AGTTCTTCCTCCAGTGTTAATTGCGG-3′ and

fordel 5′-AAT**GGGCCC**ATTGCCAATATGGCATTTACTATTGG-3′.

In bold are shown the sequences corresponding to the introduced restriction sites, AscI (HxuA^Glu713Ala^ and HxuA^Asp726Ala^ mutants), PspOMI (HxuA^DEL^ mutant). After amplification, the PCR reaction was digested with DpnI, and the PCR product digested either with AscI (HxuA^Glu713Ala^ and HxuA^Asp726Ala^ mutants) or PspOMI (HxuA^DEL^ mutant) and self-ligated before transformation into *E. coli* XL1-Blue strain. After checking for the presence of the desired mutation by sequencing, a 900-bp BsrGI-SphI fragment encompassing the desired mutation was recloned into an otherwise wild-type *hxuCBA* background. To obtain the dm variant of the mutants (that is, cysteine-less at the C-terminus), the 500-bp Bpu10I-SphI fragment from the dm variant was exchanged with that of the previously constructed plasmids (Supplementary Fig. 6).

### HxuA detection in whole cells

Polyclonal antibodies directed against full-length purified HxuA were raised in rabbits at the Institut Pasteur facility in 2011, in compliance with good practice in use at that time. The serum was used in western blots at a 1/2000 dilution after depletion of unspecific antibodies from the serum by adsorption on a cell lysate from a strain not expressing HxuA. Briefly, 20 μl of serum were mixed for 1 h at 4 °C with 1 ml of a cell lysate obtained by sonication of the equivalent of 100 ml of an overnight culture resuspended in PBS. After centrifugation (15,000*g* for 15 min), the supernatant represents the ‘depleted serum'. Blotting itself after gel electrophoresis was carried out for 2 h in a semi-dry apparatus, at 1 mA cm^−2^ and 12 V maximum on Protran BA 83 nitrocellulose membrane. Whole cultures were TCA-precipitated (10%) and the samples were run on commercial gels (mini Protean TGX 4-15% gradient gels from BioRad). The equivalent of 0.05 OD_600nm_ (ca. 10^7^ cells) was deposited on each lane.

### Haemopexin biotinylation and dot-blot assays on whole cells

Full-length haemopexin was biotinylated with the EZ-LINK Sulfo-PEG4-Biotinylation Kit from Pierce, following the kit instructions. There were 4,5 biotin molecules/haemopexin molecule, as determined using the HABA/avidin ((2- [4'-hydroxy-benzeneazo] benzoic acid)) reagent present in the kit. After haem-loading, this biotinylated haem-haemopexin was fully functional in haem acquisition tests *in vivo* (Fig. 5c, bottom). This biotinylated haemopexin at 0.1 μM was subsequently used in dot-blot assays on whole-cells expressing HxuA and its mutants in a protocol similar to that used to detect HasA-HasR interaction^51^. Briefly, cells from various strains were grown in the presence of arabinose (40 mg l^−1^) to induce the expression of the *hxu* genes up to an OD_600nm_ of 1 and 0.05 ml were spotted in duplicate for each culture on Protran BA 83 nitrocellulose membrane. After membrane drying and saturation with TBST (10 mM Tris-HCl pH 8.0, 150 mM NaCl, 0.05% Tween-20) containing 0.5% skimmed milk, the membrane was incubated for 1 h with 0.1 μM biotinylated haemopexin. Bound haemopexin was revealed using High Sensitivity Streptavidin HRP from Pierce (ref 21130).

### Biological activity tests

The strain *E. coli* C600Δ*hemA*Δ*tonB*Δ*exbBD*(ptonBHi) was transformed with the wild-type and mutant phxuCBA plasmids, and tested for overnight growth with haem and haem-haemopexin as a haem source as described previously^12^. Briefly, cells were grown up to an OD_600nm_ of 1 and 0.1 ml of cells were mixed with 3.5 ml of molten soft-agar (0.6% in lysogeny broth medium) and poured on the top of a petri dish containing lysogeny broth agar with the appropriate antibiotics and arabinose (40 mg l^−1^) to induce the expression of the *hxu* genes. Wells were made with the large end of a Pasteur pipette after solidification of the top agar, and filled with 100 μl of the various haem sources, at 5 μM. Growth around the wells was scored after overnight incubation at 37 °C.

## Additional information

**Accession codes:** HxuA structure and HxuA-NtHpx structure have been deposited in the PDB under the accession codes 4RM6 (HxuA) and 4RT6 (HxuA-NtHpx).

**How to cite this article**: Zambolin, S. *et al.* Structural basis for haem piracy from host haemopexin by Haemophilus influenzae. *Nat. Commun.* 7:11590 doi: 10.1038/ncomms11590 (2016).

## Supplementary Material

Supplementary Information Supplementary Figures 1-6 and Supplementary Tables 1-4.

## Figures and Tables

**Table 1:** HxuA data collection and refinement statistics.

	**HxuA**
*Data collection*	
Space group	P2_1_2_1_2
Cell dimensions	
*a*, *b*, *c* (Å)	93.40, 177.13, 54.94
α, β, γ (°)	90.00, 90.00, 90.00
Resolution (Å)	1.60 (1.65-1.60)
*R*_merge_	6.6 (124.4)
*I*/σ*I*	12.71 (1.74)
Completeness (%)	98.8 (98.0)
Redundancy	5.6 (5.5)
	
*Refinement*	
Resolution (Å)	1.60
No. reflections	119,491
*R*_work_/*R*_free_	0.188/0.211
No. atoms	
Protein	6,026
Ligand/ion	/
Water	737
B-factors	
Protein	34.25
Ligand/ion	–
Water	46.78
R.m.s. deviations	
Bond lengths (Å)	0.010
Bond angles (°)	1.16

Values in parentheses are for the highest resolution shell.

**Table 2:** HxuA-NtHpx data collection and refinement statistics.

	**HxuA-NterHpx**
*Data collection*	
Space group	P2_1_2_1_2
Cell dimensions	
*a*, *b*, *c* (Å)	45.06, 348.86, 77.35
α, β, γ (°)	90.00, 90.00, 90.00
Resolution (Å)	2.80 (2.97-2.80)
*R*_merge_	11.5 (67.9)
*I*/σ*I*	13.72 (2.14)
Completeness (%)	96.3 (85.2)
Redundancy	7.23 (5.15)
	
*Refinement*	
Resolution (Å)	2.80
No. reflections	29,966
*R*_work_/*R*_free_	0.18/0.23
No. atoms	
Protein	7,621
Ligand/ion	28
Water	192
B-factors	
Protein	55.70
Ligand/ion	119.92
Water	39.55
R.m.s. deviations	
Bond lengths (Å)	0.010
Bond angles (°)	1.22

Values in parentheses are for the highest resolution shell.
