# Supplementary material for: Structural basis for haem piracy from host haemopexin by Haemophilus influenzae
Source: Nat Commun. 2016 May 18;7:11590. doi: 10.1038/ncomms11590 (PMC4873976; doi:10.1038/ncomms11590)
Supplement: Supplementary Information — Supplementary Figures 1-6 and Supplementary Tables 1-4. [file ncomms11590-s1.pdf]

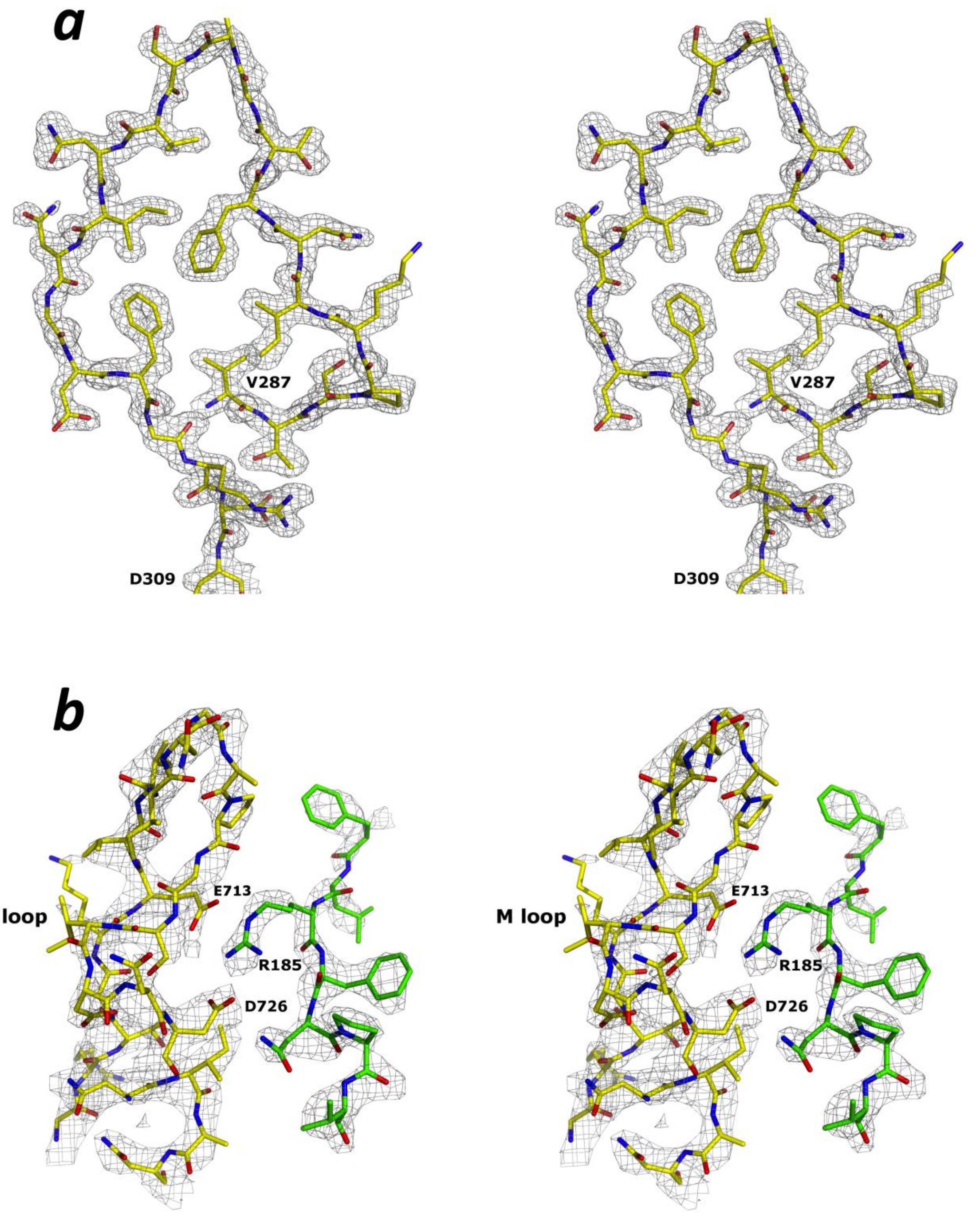

Supplementary Figure 1: Stereo views for HxuA (a) and the HxuA-NtHpx complex (b). Stereo views of composite simulated annealing omit maps covering residues 287 to 309 of HxuA (a) and the interface between the M loop of HxuA (residues Gly708-Asn731) and residues F183-V189 of NtHpx (b). HxuA and NtHpx are colored in yellow and green, respectively. The electron density maps are contoured at the 1,5 σ level.

Purified HxuA

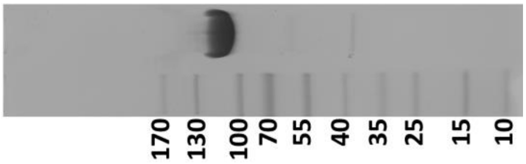

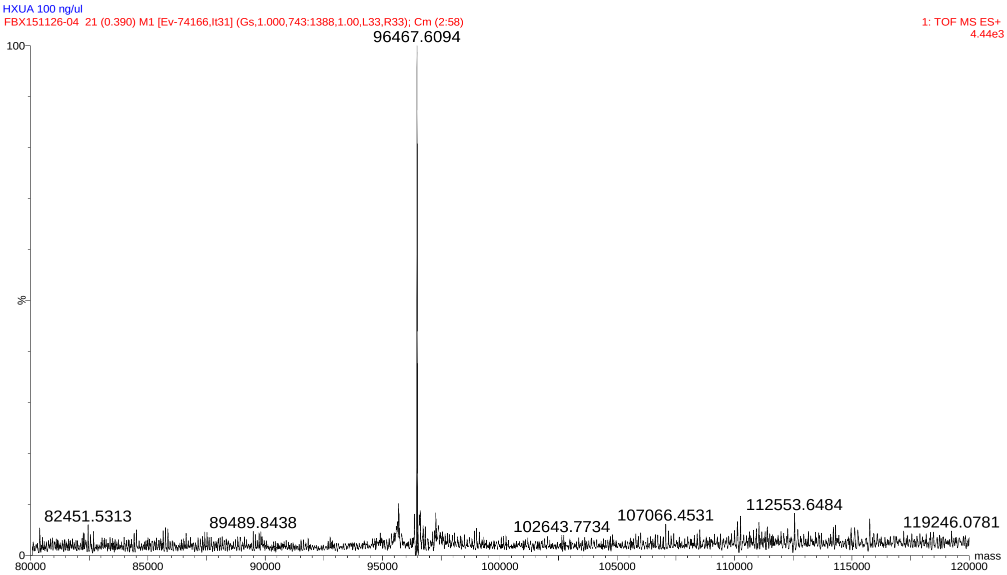

HxuA crystal

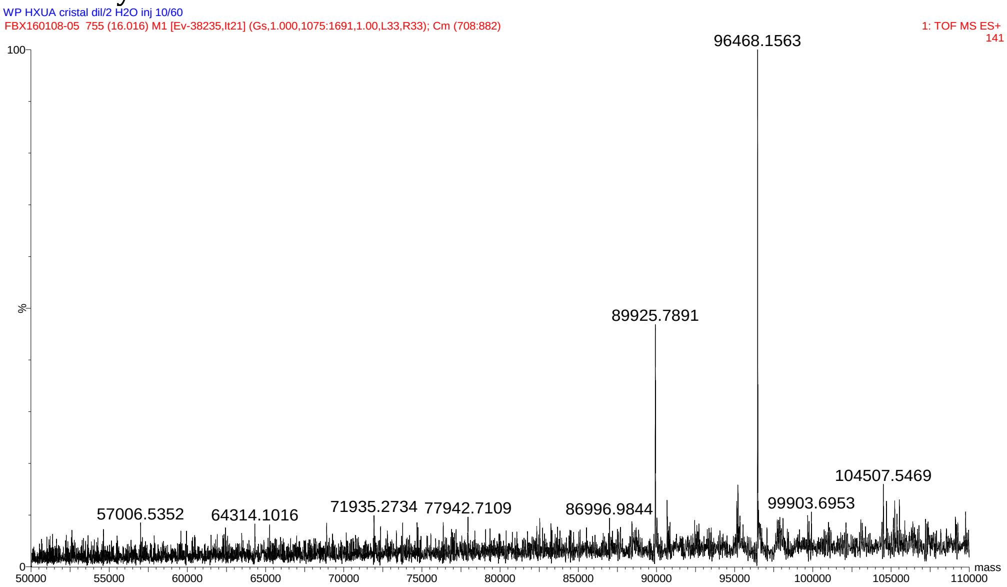

Supplementary Figure 2a : Analysis of purified HxuA and of a crystal of HxuA. The top panel shows a stained gel of purified HxuA together with a molecular weight marker scale (in kDa). HxuA does not migrate at its correct molecular weight in SDS-PAGE. The faint band just below the 40kDa represents a minor contaminant partially overlapping HxuA in the mono Q step. The two other panels show mass spectrometry analysis of purified HxuA and of a redissolved crystal. The theoretical molecular weight of HxuA is 96469.3047 Da. The samples were analyzed on a Q-TOF Micro mass spectrometer from Waters.

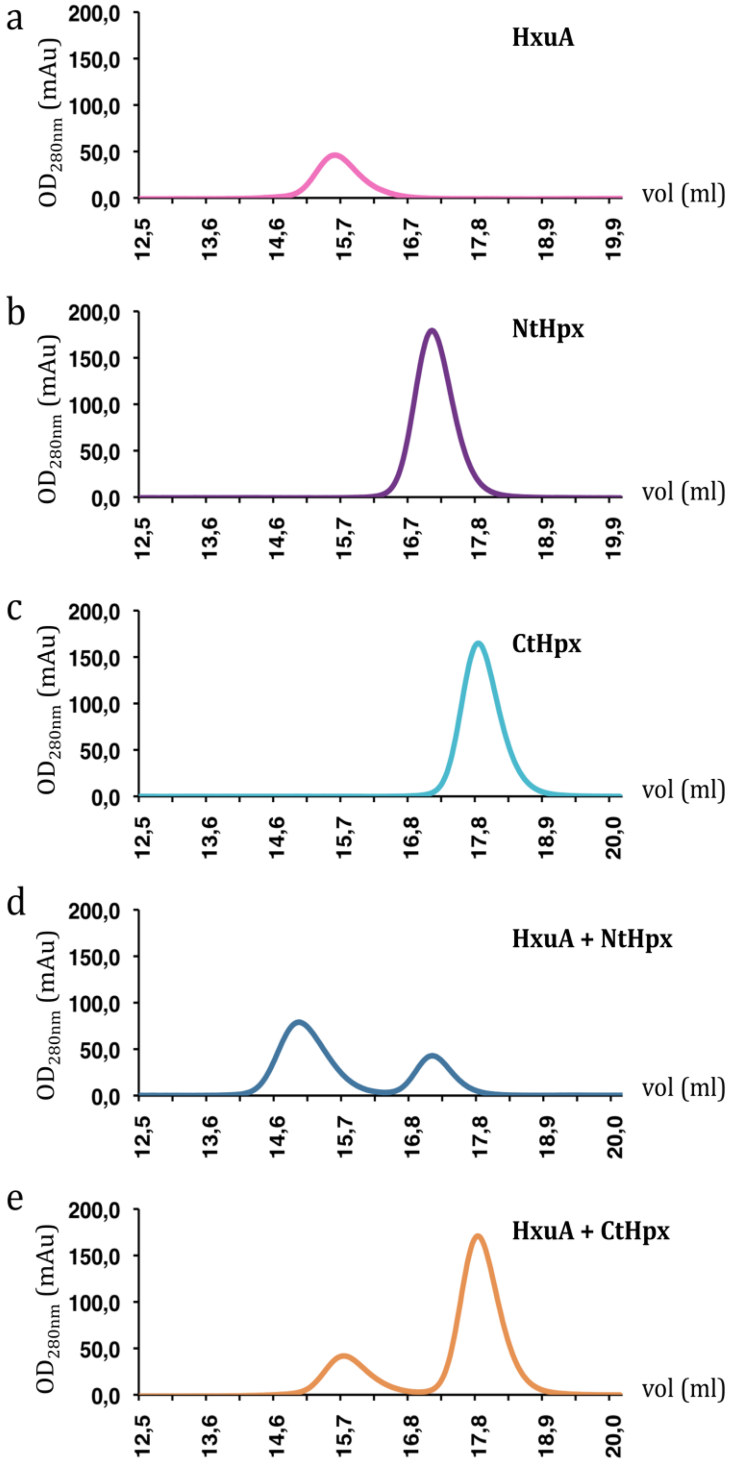

Supplementary Figure 2b: Size exclusion chromatography analysis of HxuA (panel a), NtHpx (panel b), CtHpx (panel c), HxuA+NtHpx (panel d) and HxuA+CtHpx (panel e). For each experiment ca. 200 μl of purified protein at a concentration of 5-10 μM were analyzed on a superose 6 column (10/300, 24 ml) from Pharmacia, in 20 mM Tris pH 7,5 10 mM EDTA 150 mM NaCl, at a flow rate of 0,4 ml min^{-1} at 15 °C. Detection was at 280 nm. For the HxuA+NtHpx and HxuA+CtHpx cases, a ca. twofold molar excess of the hemopexin fragment with respect to HxuA was loaded.

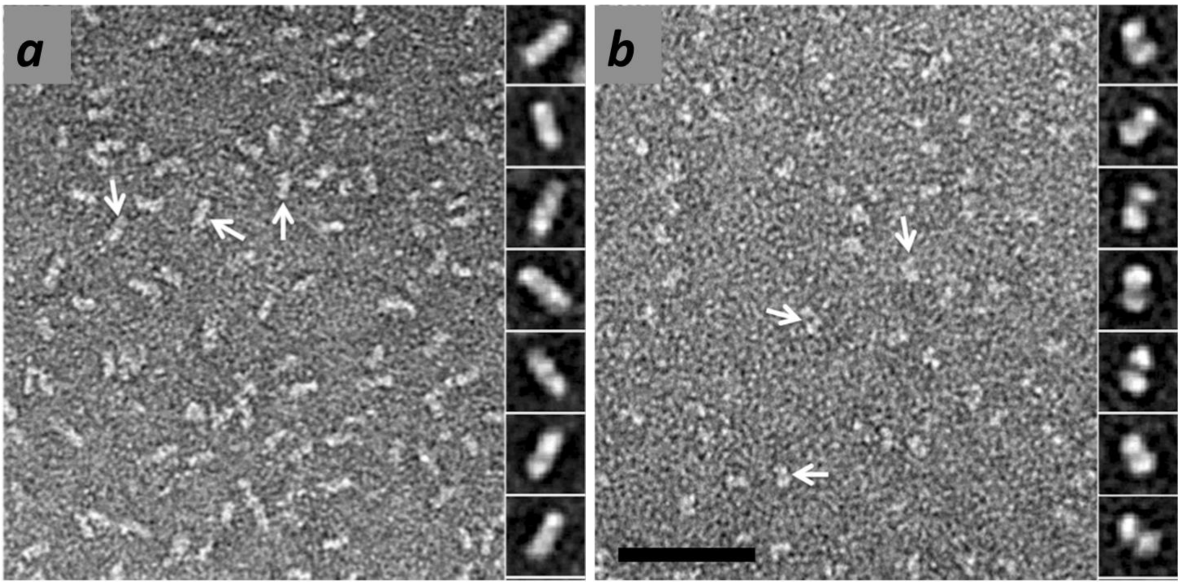

Supplementary Figure 3: TEM analysis of Hemopexin and HxuA

a: Electron micrograph of negatively stained HxuA. The arrows indicate the protein particles adsorbed on the carbon film. The right panel shows a representative gallery of class averages from 4000 particle projections analyzed by the EMAN2 software. **b**: Electron micrograph of negatively stained hemopexin. The arrows indicate the protein particles adsorbed to the carbon film. The right panel shows a representative gallery of class averages from 3677 particle projections analyzed by the EMAN2 software. The scale bar is 50 nm. All inset boxes are 18 nm wide.

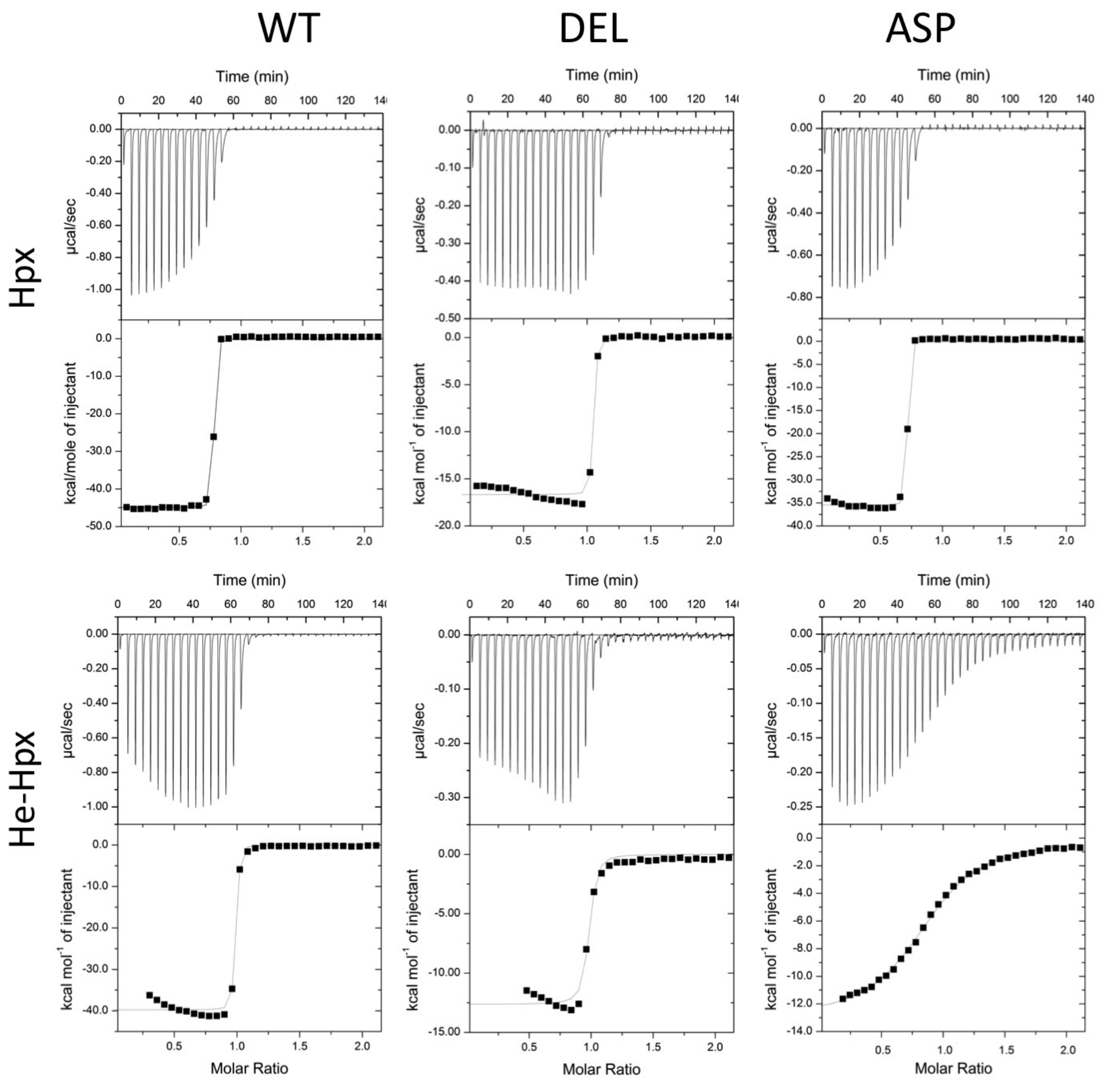

Supplementary Figure 4: Interaction of HxuA (left), HxuA^{DEL} (center) and HxuA^{Asp726Ala} (right) with hemopexin (top) and heme-hemopexin (bottom), as measured by ITC. For each experiment, the upper part shows the heat signal for the titration and the lower part shows the binding isotherm derived from the heat signal, together with the fit calculated with Origin software.

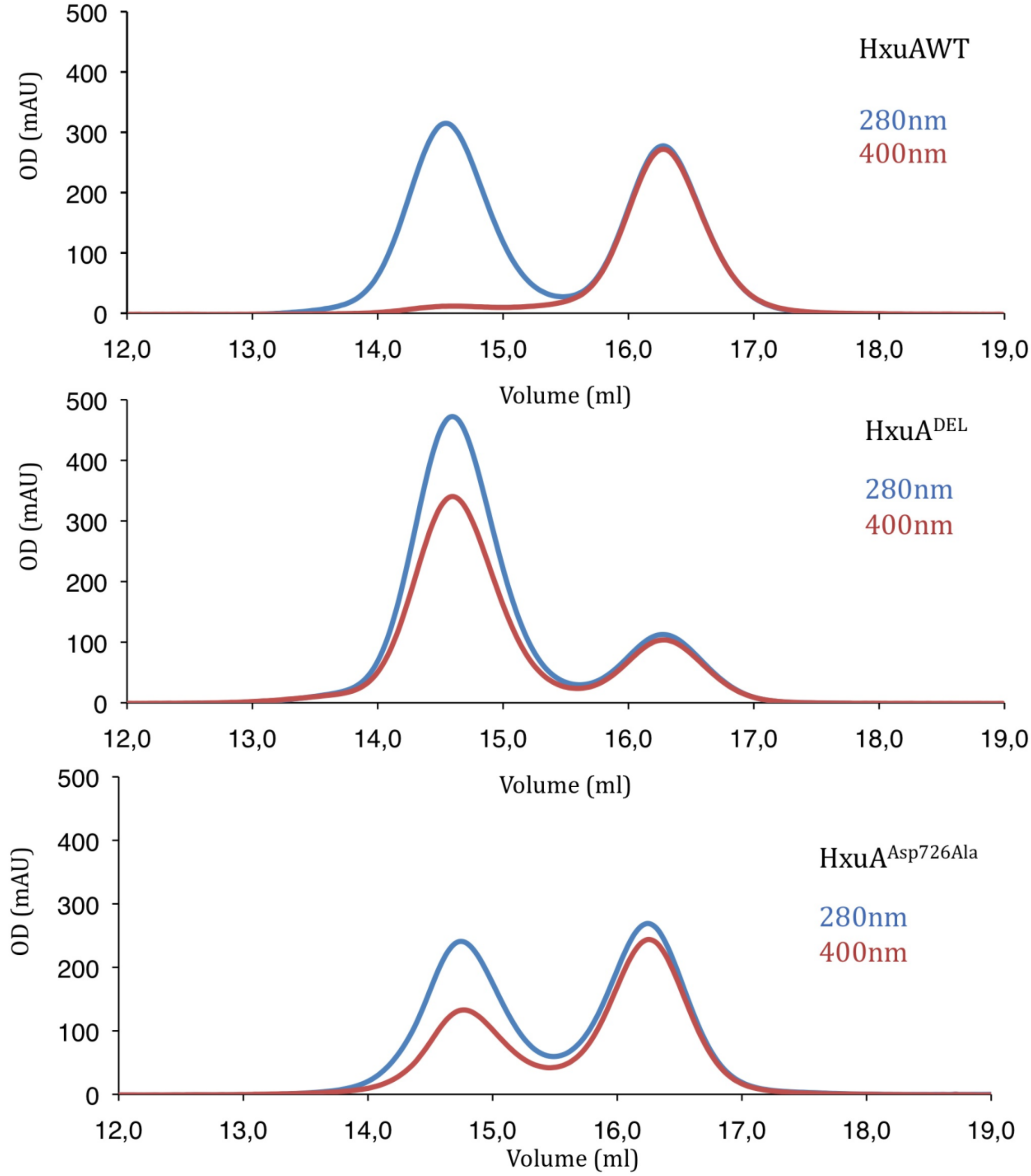

Supplementary Figure 5a: Size exclusion chromatography analysis of the complexes between a ca. twofold excess of heme-hemopexin with HxuA, HxuA^{DEL}, HxuA^{Asp726Ala}. Absorbances at both 280 nm (blue) and 410 nm (red) were recorded. The peak at around 16,2 ml contained free He-Hpx, the other peak contained the complex.

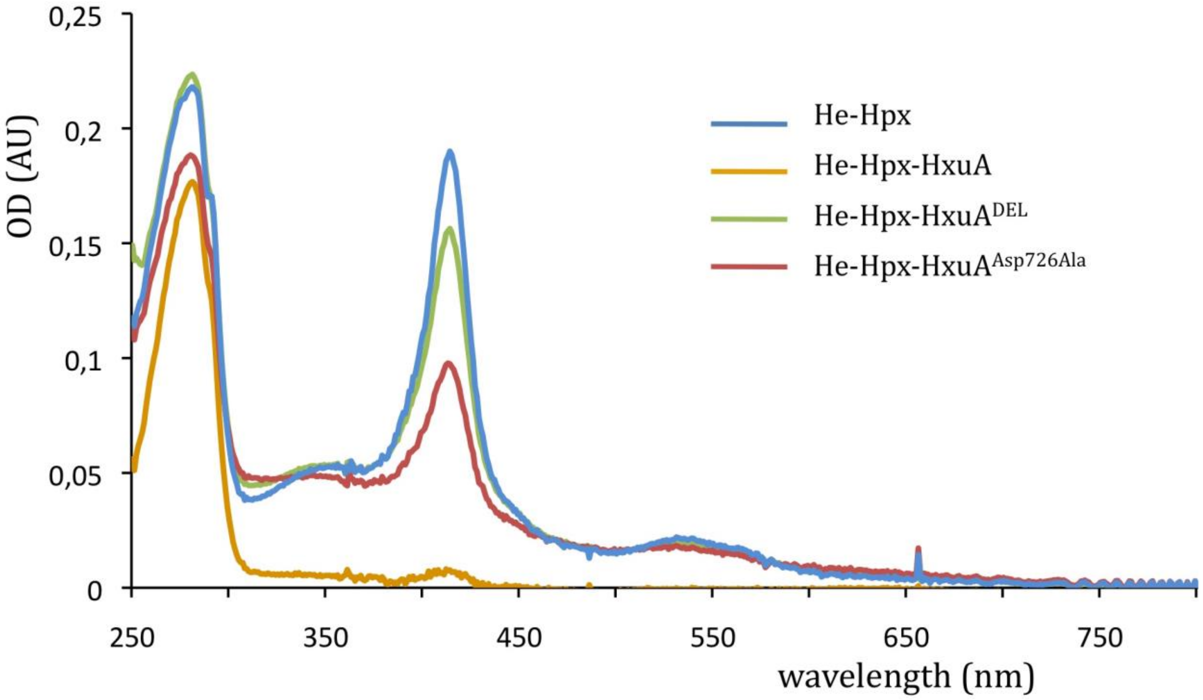

Supplementary Figure 5b: Absorption spectra of the three complexes and of heme-hemopexin.

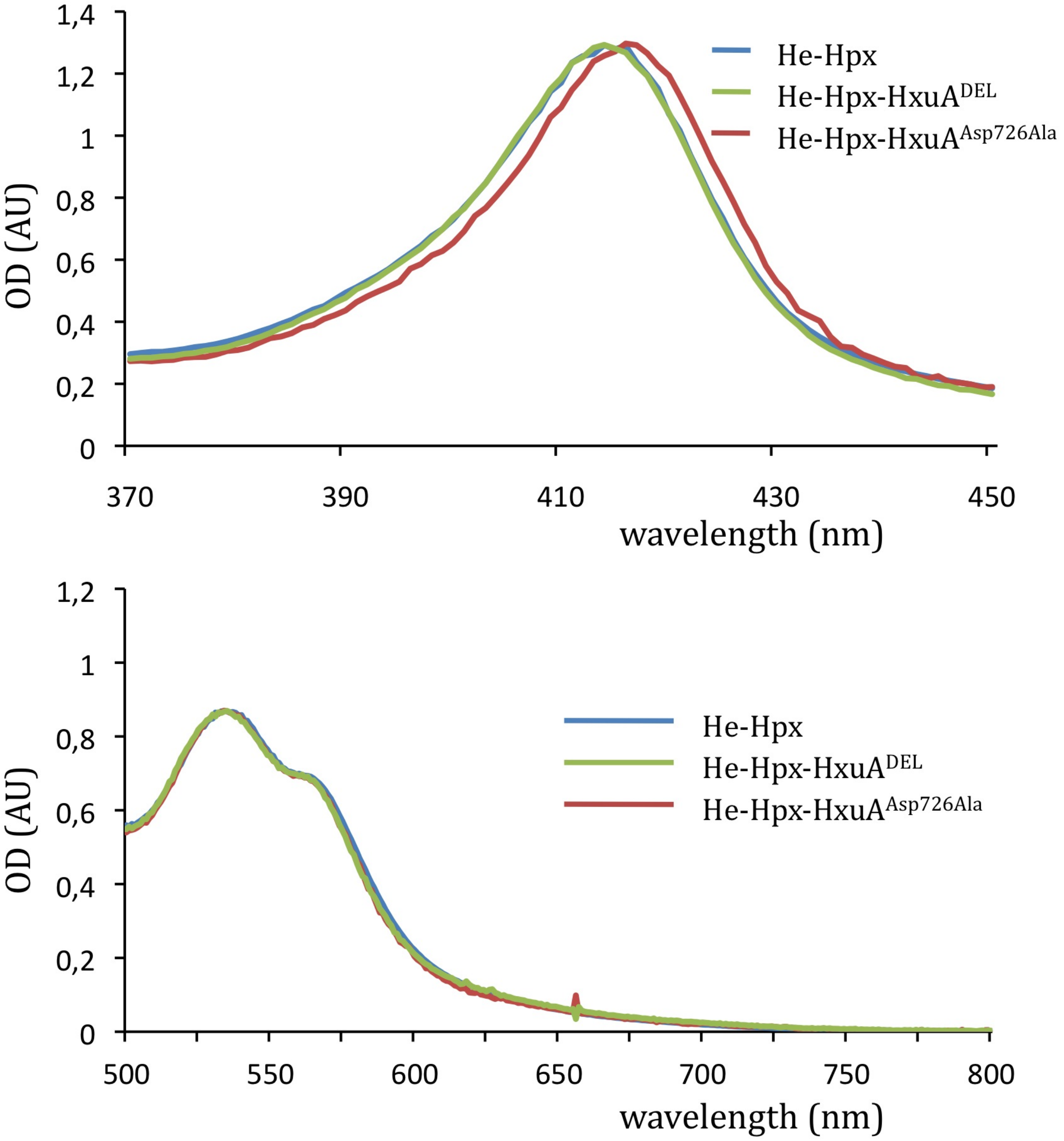

Supplementary Figure 5c: Enlargment of the spectra from Supplementary Figure 5b in the Soret band (top panel) and in the α and β region (bottom panel). Spectra were normalized on the local maximum, and recorded on concentrated samples.

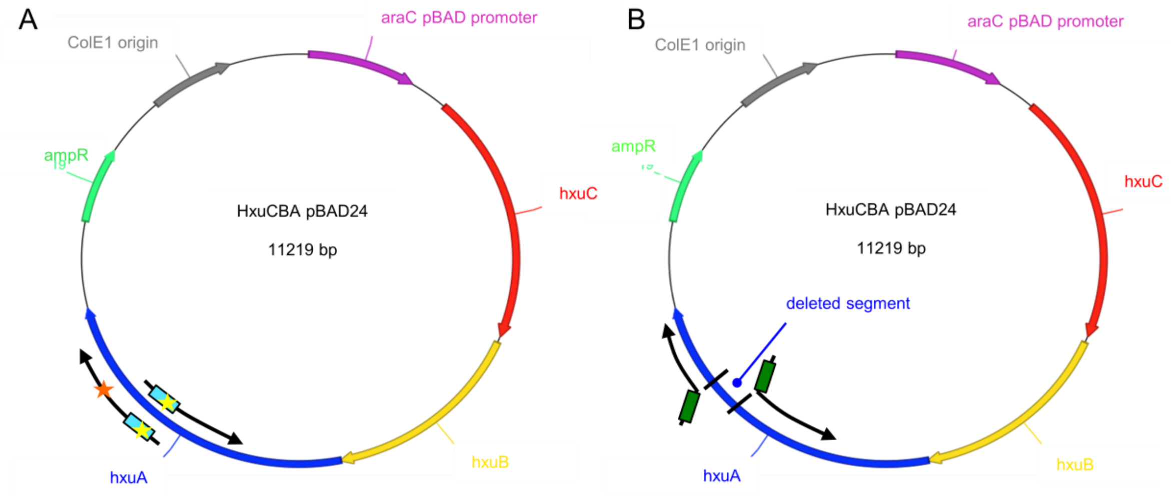

Supplementary Figure 6: cloning strategy for obtaining the mutants. The plasmid used to obtain the mutants is represented as a circle, with its characteristic elements. The *hxuA* gene is represented in blue. A: the Asp726Ala mutation is represented by an orange star; the yellow stars represent the introduced silent mutation corresponding to the AscI restriction site. The same strategy was used for the Glu713Ala mutant. B: The green cassette represents the added PspOMI restriction site encompassing the deleted fragment. This restriction site leads to an additional Pro codon in the final sequence, replacing the initial 712-728 sequence.

Supplementary Table 1: Secondary structures in HxuA

Strands in β-helix:

Parallel β-sheet 2	parallel β-sheet 1	parallel β-sheet 3
PB2	PB1	PB3
	β2(Ala16-Ile21)	β1(Ser10-Val13)
	β3(Lys24-Gln29)	β4(Thr33-Trp38)
β5(Ser40-Ile43)	β6(Glu48-Lys52)	β7(Val59-Val64)
β8(Ser70-Ile72)	β9(Lys75-Ala78)	β10(Lys81-Ala85)
β11(Val90-Ile92)	β12(Glu97-Asn99)	β13(Gly102-Thr106)
β14(Gln138-Asn141)	β15(Lys144-Lys146)	β16(Phe150-Asn154)
β17(Lys157-Asn160)	β18(Glu163-Asp165)	β19(Lys171-Ser176)
β22(Ile201-Asn204)	β23(Ile207-Ala210)	β24(Asp212-Ala217)
β25(Leu227-Asn230)	β26(Val233-Ala236)	β29(Lys245-Ser249)
β30(Asp252-Leu255)	β31(Asp260-Gly263)	β32(Glu266-Thr270)
β33(Lys276-Ser281)	β34(Lys286-Thr288)	β35(Lys291-Thr295)
β36(Ser298-Gly303)		β39(Glu324-Asp328)
β41(Ser351-Gln354)		β42(Lys367-Leu370)
β40(Ile335-Ala338)		
β43(Val375-Ser378)	β44(Gly379-Ile383)	β45(Ser391-Leu395)
β46(Ile401-His405)	β47(Ala406-Ser410)	β48(Leu414-Ile417)
β49(Ile430-Asp434)	β50(Ser435-Leu439)	β51(Gly440-Gly447)
β52(Phe471-Asn477)	β53(Val478-Gln482)	β54(Val483-Ala489)
β55(Phe492-Asp498)	β56(Asn499-Ala502)	β57(Asn507-Asp511)
β60(Glu579-Ser583)	β61(Lys586-Lys590)	β62(Phe593-Glu599)
β63(Lys600-Asp604)	β64(Lys607-Phe612)	β65(Leu627-Gly631)
β66(Lys632-Val636)	β67(His639-Gly644)	β68(Ala657-Gly663)
β69(Glu664-Glu668)	β70(Ser670-Ser676)	β71(Tyr680-Thr684)
β72(Lys689-Gly692)	β73(Leu699-Asn705)	β74(Met732-Ile736)
β75(Thr742-Glu747)	β76(Asp750-Leu754)	β77(Thr760-Ser764)
β78(Val768-Val772)	β79(Asn777-Phe781)	
	β80(Thr799-Lys800)	

Extra elements of secondary structures :

- Helices -

H1(Leu355-Asn364)
H2(Asp451-Cys455)
H3(Leu528-Leu533)
H4(Asp549-Met564)
H5(Asp619-Gln623)
H6(Ser802-Asn814)

- Strands –

β20(Tyr178-Phe182) / β21(Ser187-Val191)
β27(Lys238-Ser240) / β28(Lys242-Gly244)
β37(Tyr314-Asn316) / β38(Arg320-Asp322)
β58(Val514-Asn517) / β59(Ser519-Tyr523)

Only present in HxuA in complex with hemopexin:

β73’ (Glu713-Ile716)/β73’’ (Ala719-Ser722)

Supplementary Table 2: Polar bonds stabilizing M loop conformation in HxuA structure.

M-loop	Distance [Å]	HxuA	Secondary structure
Thr 706[N]	2,96	Ser 676[O]	β70
Thr 706[OG1]	3,36	Ser 676[O]	β70
Arg 709[NH2]	3,70	Leu 783[O]	Loop β79-β80
Arg 709[NH2]	3,43	Pro 784[O]	Loop β79-β80
Ile 716[N]	2,87	Tyr 680[OH]	β71
Asn 717[ND2]	3,06	Asp 738[O]	Loop β74-β75
Asn 717[ND2]	3,45	Ser 765[OG]	Loop β77-β78
Thr 706[O]	2,90	Asn 757[ND2]	Loop β76-β77
Thr 706[OG1]	2,76	His 677[ND1]	Loop β70-β71
Gly 707[O]	3,78	Gly 758[N]	β68
Gly 707[O]	3,27	Gly 759[N]	β68
Glu 713[OE2]	2,72	Thr 760[OG1]	β68
Val 714[O]	2,71	Tyr 680[OH]	β71
Ile 716[O]	2,83	Arg 683[NH1]	β71
Asn 717[OD1]	3,84	Asp 738[N]	Loop β74-β75
Asn 717[OD1]	3,11	Arg 683[NH2]	β71
Pro 720[O]	3,48	Asn 555[ND2]	Helix H4
Asn 731[O]	2,85	Thr 760[N]	β77
Asn 731[OD1]	2,96	Gly 679[N]	Loop β70-β71

M loop residues are numbered, and the interacting residues are shown, together with the distances and the secondary structure elements to which they belong.

Supplementary Table 3: Thermodynamic parameters from ITC experiments.

	**WT-Hpx**	**WT-HeHpx**	**Del-Hpx**	**Del-HeHpx**	**Asp-Hpx**	**Asp-HeHpx**	**WT-NtHpx**
N	0,8 (0,05)	0,96	1,07 (0,05)	0,89 (0,08)	0,68 (0,01)	1,06 (0,17)	0,88
K (M^{-1})	> 10^9	> 10^9	> 10^9	$1{,}7x10^8$ (0,25)	> 10^9	$2{,}9x10^6$ (1,3)	$7{,}6x10^7$
ΔH (kcal mol^{-1})	-50,2 (3,8)	-39,8	-16,5 (0,5)	-13,0	-37,2 (2)	-12,8 (0,5)	-48,5
ΔS (cal mol^{-1} K^{-1})	-124 (11,3)	-92	-12,7 (0,5)	-5,8 (0,07)	-83 (7)	-13,5 (1)	-127

N represents the stoichiometry, *K* the affinity constant (M^{-1}), ΔH the enthalpy variation (kcal mol^{-1}) and ΔS the entropy variation (cal mol^{-1} $°C^{-1}$). The results represent the mean of two experiments, except for the WT-Nter, the WT-He-Hpx cases (one experiment) and the WT-Hpx (three experiments). In the cases where two or three experiments have been carried out, one was with a 8 minutes delay between each injection. Values in parentheses represent the error on the mean. K values above 10^9 M^{-1} are indicated > 10^9. For each column, the interactants are in bold, WT, Del and Asp respectively refer to HxuA, $HxuA^{DEL}$ and $HxuA^{Asp726Ala}$, whereas Hpx, He-Hpx and NtHpx respectively refer to hemopexin, heme-hemopexin and the N-terminal domain of hemopexin.

Supplementary Table 4: Interface of the HxuA molecule with NtHpx in the crystal structure.

NterHpx secondary structures	NterHpx - residues	Distance [Å]	HxuA secondary structures	HxuA residues
**Blade 3** - junction between strands 1 and 2	Arg 128[NE]	2,77	Helix H4	Asp 551[OD2]
	Arg 128[NH2]	2,83		Asn 548[O]
	Arg 128[NH2]	2,91		Asp 551[OD1]
	Gln 132[NE2]	2,85		Asn 555[OD1]
	Gln 132[O]	3,22		Asn 555[ND2]
	Asp 133[OD2]	3,47		Asn 555[ND2]
	Gln 132[NE2]	3,69	Loop β67-β68	Tyr 656[OH]
**Blade 3** - junction between strands 3 and 4	Thr 150[O]	2,93	Loop β51-β52	Arg 462[NH1]
	Thr 151[O]	3,40		Arg 462[NH2]
**Blade 3** - strand 4	Lys 155[NZ]	2,77	Helix H4	Asp 563[O]
	Lys 155[NZ]	3,17		Asp 563[OD1]
	Arg 157[NH2]	2,66		Asp 563[OD2]
	Glu 156[N]	2,91	Loop β57-β60	Asn 569[OD1]
	Lys 155[NZ]	3,00	Loop β71-β72	Asp 685[OD1]
	Lys 155[NZ]	2,86		Asp 685[OD2]
	Lys 155[NZ]	3,76	Loop β74-β75	Asp 738[OD2]
	Ser 158[N]	2,65		Asp 738[O]
	Glu 156[O]	3,11		Ala 740[N]
Blade 4 – strand 2	Arg 174[NH1]	3,17	Strand β73' "M Loop"	Ile 716[O]
	Arg 174[NH2]	3,16		Ile 716[O]
	Arg 174[NH1]	3,00	Strand β73'' "M Loop"	Ala 719[O]
	Arg 174[NH1]	3,21		Pro 720[O]
	Tyr 176[OH]	2,90		Pro 720[O]
**Blade 4** – strand 3	Arg 185[NE]	2,67	Strand β73' "M Loop"	Glu 713[OE1]
	Arg 185[NH2]	3,39		Glu 713[OE2]
	Arg 185[NH1]	3,15	Loop β73'' – β74	Asp 726[OD2]
	Arg 185[NH2]	2,81		Asp 726[OD1]
**Blade 4** - junction between strands 3 and 4	Ser 190[O]	3,83	β71	Tyr 680[OH]
	Tyr 197[OH]	3,36	Strand β73' "M Loop"	Glu 713[OE1]
	Asn 187[ND2]	2,81	Loop β73'' – β74	Asp 726[OD2]
	Glu 192[OE1]	2,81	Loop β79-β80	Lys 787[NZ]
	Glu 192[OE1]	2,77		Asn 788[ND2]

The NtHpx residues are shown together with the secondary structure elements they belong to, as well as the interacting residues in HxuA with their associated secondary structure element. Distances (in Å) between donors and acceptors are shown in the central column.
